# Effect of cerulenin on fatty acid composition and gene expression pattern of DHA-producing strain *Colwellia psychrerythraea* strain 34H

**DOI:** 10.1186/s12934-016-0431-9

**Published:** 2016-02-06

**Authors:** Xia Wan, Yun-Feng Peng, Xue-Rong Zhou, Yang-Min Gong, Feng-Hong Huang, Gabriel Moncalián

**Affiliations:** Key Laboratory of Biology and Genetic Improvement of Oil Crops, Ministry of Agriculture, Oil Crops Research Institute of Chinese Academy of Agricultural Sciences, Wuhan, 430062 China; Hubei Key Laboratory of Lipid Chemistry and Nutrition, Wuhan, 430062 China; CSIRO Agriculture, Canberra, ACT 2601 Australia; CSIRO Food and Nutrition, Canberra, ACT 2601 Australia; Departamento de Biología Molecular e Instituto de Biomedicina y Biotecnología de Cantabria (IBBTEC), Universidad de Cantabria-CSIC, C/Albert Einstein 22, 39011 Santander, Spain

**Keywords:** Cerulenin, DHA, *Colwellia psychrerythraea*, Fatty acid profile, RNA-seq

## Abstract

**Background:**

*Colwellia psychrerythraea* 34H is a psychrophilic bacterium able to produce docosahexaenoic acid (DHA). Polyketide synthase pathway is assumed to be responsible for DHA production in marine bacteria.

**Results:**

Five *pfa* genes from strain 34H were confirmed to be responsible for DHA formation by heterogeneous expression in *Escherichia coli*. The complexity of fatty acid profile of this strain was revealed by GC and GC–MS. Treatment of cells with cerulenin resulted in significantly reduced level of C16 monounsaturated fatty acid (C16:1^Δ9t^, C16:1^Δ7^). In contrast, the amount of saturated fatty acids (C10:0, C12:0, C14:0), hydroxyl fatty acids (3-OH C10:0 and 3-OH C12:0), as well as C20:4ω3, C20:5ω3 and C22:6ω3 were increased. RNA sequencing (RNA-Seq) revealed the altered gene expression pattern when *C. psychrerythraea* cells were treated with cerulenin. Genes involved in polyketide synthase pathway and fatty acid biosynthesis pathway were not obviously affected by cerulenin treatment. In contrast, several genes involved in fatty acid degradation or β-oxidation pathway were dramatically reduced at the transcriptional level.

**Conclusions:**

Genes responsible for DHA formation in *C. psychrerythraea* was first cloned and characterized. We revealed the complexity of fatty acid profile in this DHA-producing strain. Cerulenin could substantially change the fatty acid composition by affecting the fatty acid degradation at transcriptional level. Acyl-CoA dehydrogenase gene family involved in the first step of β-oxidation pathway may be important to the selectivity of degraded fatty acids. In addition, inhibition of FabB protein by cerulenin may lead to the accumulation of malonyl-CoA, which is the substrate for DHA formation.

**Electronic supplementary material:**

The online version of this article (doi:10.1186/s12934-016-0431-9) contains supplementary material, which is available to authorized users.

## Background

Docosahexaenoic acid (DHA, 22:6ω3) and eicosapentaenoic acid (EPA, 20:5) are the two main commercial omega-3 very long chain polyunsaturated fatty acids (VLCPUFAs) because of their health benefits. Their beneficial effects in human cardiovascular health, mental health, immune function and infant cognitive development are well documented [1]. In addition, they are precursors for certain hormones and lipid-derived signaling molecules [2]. Such VLCPUFAs can be synthesized in human with a low overall conversion rate using α-linolenic acid (ALA, 18:3) as substrate [3], rather than *de novo* synthesis. Alternatively, food or food additives rich in VLCPUFAs are a good choice to obtain VLCPUFAs directly.

Commercial VLCPUFAs are mainly produced from fish oils, algae oils or krill oils. Recently, it became more and more attractive to achieve commercial production of VLCPUFAs via metabolic engineering of microbes due to the limited natural resources. Microbes grow fast, are easy to be genetically manipulated and use versatile carbon sources. One of these successful examples, oleaginous yeast *Yarrowia lipolytica* has been genetically engineered to make EPA at 25 % dry cell weight and at 50 % of total lipids, which is much higher than those of other reported wild-type microbes [4]. In general, VLCPUFAs could be synthesized through conventional fatty acid desaturase/elongase pathway in algae and animals. There are more than 20 fatty acid desaturases and fatty acid elongases involved in this process [5]. Several key desaturases and elongases, such as Δ6 desaturase, Δ5 desaturase, Δ4 desaturase, Δ9 elongase, Δ8 desaturase, Δ5 elongase, have been transformed into microbes or plants to make them successfully produce VLCPUFAs [6–8]. Alternatively, VLCPUFAs can be synthesized via unconventional polyketide synthase (PKS) pathway as well. This pathway can only be found in narrow species of marine bacteria including *Shewanella*, *Moritella*, *Vibrio*, *Photobacterium*, *Aureispira* and *Colwellia* and some lower eukaryotes [9–12]. PKS system contains a set of essential domains for VLCPUFAs formation including malonyl-CoA:ACP acyltransferase (MAT), acyl carrier protein (ACP), 3-ketoacyl synthase (KS), 3-ketoacyl-ACP reductase (KR), acyltransferase (AT), chain length factor (CLF), enoyl reductase (ER), and 3-hydroxyacyl-ACP dehydrase/isomerase (HD/IS) [13]. Compared to conventional desaturase/elongase pathway, there are only 3–5 genes (*pfa* gene cluster) responsible for *de novo* VLCPUFAs synthesis via PKS pathway [12]. This cluster usually contains 1 MAT domain (*pfaA*), 1 KR domain (*pfaA*), 1 CLF domain (*pfaC*), 1 ER domain (*pfaD*), 2 KS domains (*pfaA* and *pfaC*), 3–6 ACP domains (*pfaA*), 1 AT (*pfaB*) and 2 DH/IS domains (*pfaC*) [12]. In addition, PKS pathway was demonstrated to be anaerobic pathway and less NADPH was needed for the whole process compared with that of conventional pathway [14]. Notably, PKS proteins typically show high sequence similarity to enzymes of fatty acid synthase (FAS) but not to any desaturase or elongase [5]. Similarly to bacterial FAS, PKS make use of *pfa* gene cluster to accomplish the iterative cycle of condensation, reduction, dehydration and further reduction to produce VLCPUFAs. However, the details of synthetic route for VLCPUFAs via PKS and the mechanism of crosstalk between FAS and PKS are still unknown.

*Colwellia psychrerythraea* strain 34H, is a psychrotrophic gram-negative bacterium isolated from the sediment of Northeast Water Polynya, Greenland. The presence of a *pfa* gene cluster in the *C. psychrerythraea* genome suggested that this strain is able to produce DHA [15]. Recently, *C. psychrerythraea* 34H was demonstrated to produce 2.3 % (w/w) of DHA and trace amount of EPA to the total fatty acids [16]. Cerulenin [(2R,3S)-2,3-epoxy-4-oxo-7,10-trans,trans-dodecadienamide] is an FAS inhibitor originally developed as an antifungal antibiotic. It was reported to be an irreversible inhibitor of β–ketoacyl-ACP synthase I and II activities [17, 18] and it could increase the DHA content in *Photobacterium*, *Shewanella* and *Moritella* [19, 20]. However, the mechanism of this process is still unknown. In the present study we describe the DHA production by *C. psychrerythraea* at different temperatures and with the treatment of cerulenin. Five *pfa* genes were required for DHA production. Cerulenin treatment resulted in significant increased DHA production, along with the increased levels of medium chain, short chain saturated fatty acids (SFAs) and hydroxyl fatty acids (HFAs), at the expense of medium chain monounsaturated fatty acids (MUFAs). RNA deep sequencing revealed a certain number of genes either up regulated or down regulated, suggesting possible involvement of these genes in regulating of the fatty acid composition.

## Methods

### Chemicals, enzymes and kits

Fatty acid standards, 2-amino-2-methyl-1-propanol, 1 % trimethylchlorosilane in N,O-bis(trimethylsilyl)trifluoroacetamide (BSTFA) and cerulenin were purchased from Sigma-Aldrich (St. Louis, MO). Zwittergent, lysozyme and proteinase K were purchased from Merck, Sigma-Aldrich (St. Louis, MO) and Roche Diagnostic GmbH (Mannheim, Germany), respectively. RNA protect reagent, RNeasy Mini Kit and Qiaquick PCR spin columns were purchased from Qiagen (Qiagen, Venlo, The Netherlands). TURBO DNA-free Kit was purchased from Applied Biosystems (Foster City, CA). Ribo-Zero rRNA removal kit for Gram-negative bacteria was purchased from Epicentre. Superscript II Reverse Transcriptase, RNaseH and DNA polymerase I were purchased from Invitrogen (Waltham, MA). PrimeScript RT reagent kit with gDNA eraser and SYBR *PremixEx Taq* II kit were purchased from TaKaRa (Dalian, Japan).

### Strains and growth conditions

The marine psychrophile bacterium *C. psychrerythraea* 34H was purchased from ATCC. This strain was cultured in marine broth 2216 (Difco Laboratories) at the temperature ranging from 0 to 15 °C without shaking. For solid media, agar was added at 15 g/L. Cerulenin dissolved in 50 % (v/v) ethanol was added to culture medium at various concentrations prior to cultivation. To investigate the effect of cerulenin on *C. psychrerythraea*, cells were cultivated at 10 °C with shaking at 200 rpm for 24 h after cerulenin added.

### Fatty acid profile analysis

*Colwellia psychrerythraea* 34H cells were harvested and freeze-dried overnight. Fatty acid methyl esters (FAME) were prepared by direct trans-methylation method [8]. The FAMEs were analyzed by GC-FID (7890A GC, Agilent Technologies, Palo Alto, CA, USA) equipped with a 30 m BPX70 column (0.25 mm inner diameter, 0.25 μm film thickness, SGE, Austin, Texas, USA). Peaks were integrated with Agilent Technologies ChemStation software. GC–MS analysis was essentially performed according to Zhou et al. [21]. Dimethyloxazoline (DMOX) derivatives of FAMEs were prepared according to Fay and Richli [22] to determine the double bond positions. For hydroxyl fatty acids, the FAMEs were further derivatized by trimethylchlorosilane as described [21] to make trimethylsilyl (TMS) derivatives.

### Heterogeneous expression of *pfa* gene cluster in *Escherichia coli*

The primers used in this section are listed in Additional file 1: Table S1. Long PCR was carried out with the high fidelity PrimeSTAR GXL DNA Polymerase (Takara Bio) according to manufacturer’s instruction. Specifically, the *C. psychrerythraea pfaABCD* sequence was amplified with primers mpfaA-F containing *EcoR*I restrction site and mpfaD-R containing *Sal*I restrction site, respectively. The resulting DNA fragment of approximately 19 kbp was double digested with *EcoR*I and *Sal*I, and then subcloned into the pColdI (Takara Bio) to generate pColdI-*pfa*A-D. *pfaE* was amplified with primers mpfaE-F containing *Kpn*I restriction site and mpfaE-R containing *BamH*I restriction site, respectively. The resulting sequence of about 900 bp was double digested with *Kpn*I and *BamH*I. Then it was inserted into pSTV28 (Takara Bio) to form pSTV28-*pfa*E. Two compatible vectors were co-transformed into *E. coli* DH5α by electroporation. Positive colonies were confirmed by PCR and sequencing. Transformants were then cultivated in Luria–Bertani (LB) medium with 50 mg/L ampicillin and 17 mg/L chloramphenicol. Half mL of cells pre-cultured at 30 °C was inoculated into 50 mL of fresh LB medium supplemented with 50 mg/L ampicillin and 17 mg/L chloramphenicol. When OD_600_ reached at about 0.6, 1 mM isopropyl β-_D_-1-thiogalactopyranoside (IPTG) was added into the culture. Then cells were cultivated with shaking at 180 rpm at 15 °C for 24 h to allow gene expression. Cells were collected and dried.

### RNA extraction, RNA enrichment and preparation of cDNA fragment libraries

*Colwellia psychrerythraea* 34H was grown in 2216 medium at 10 °C. Overnight grown cells were inoculated into culture flasks containing 3 mL of 2216 media each, under two treatments with three repeats, One was cultured at 10 °C (M10), while the other treatment also cultured at 10 °C but added cerulenin at a final concentration of 12 μg/mL (M10_C). *Colwellia psychrerythraea* cells were collected at exponentially growing phase (typical optical density at 600 nm~0.9), then fixed with two volumes of RNA protect reagent and harvested by centrifugation. The cellular pellet was then lysed with 280 μL of lysis buffer (10 % zwittergent, 15 mg/ml lysozime and 20 mg/ml proteinase K in TE buffer). Total RNA was isolated using the RNeasy Mini Kit according to manufacturer’s instructions. During this RNA purification, the residual DNA in the samples was removed using TURBO DNA-free Kit. NanoDrop ND-1000 spectrophotometer (NanoDrop Technology, Rockland, DE) and Experion Automated Electrophoresis were used to check the quantity and quality of RNA with the RNA StdSens Analysis Kit (Bio Rad). Genomic DNA contamination was tested by quantitative real-time PCR (qPCR) amplification of 16S rRNA using the iO5 iCycler (Bio-Rad). Total RNA samples were stored at −80 °C until further use.

Ribo-Zero rRNA Removal Kit was used to remove 16S and 23S rRNA from total RNA according to the manufacturer’s protocol with the exception that no more than 5 μg total RNA was treated per enrichment reaction. Each RNA sample was divided into multiple aliquots of ≤5 μg RNA and separate enrichment reactions were performed for each sample. Enriched mRNA samples were pooled and analyzed on the 2100 Bioanalzyer (Agilent) to asses the reduction of 16S and 23S rRNA before the preparation of cDNA fragment libraries. 100 ng of mRNA were treated with the Ambion RNA fragmentation reagents to generate 60-200 nucleotide RNA fragments. After fragmented RNA precipitation, the synthesis of the cDNA first strand was performed using random N6 primers and Superscript II Reverse Transcriptase. Second strand cDNA synthesis followed, using RNaseH and DNA Polymerase I. Double stranded cDNA was purified using Qiaquick PCR spin columns according to the manufacturer’s protocol.

### Illumina Genome Analyzer RNA-Seq

This double-stranded cDNA was then processed for RNA-Seq using the Illumina Genomic DNA Sample Prep kit (Illumina, Inc., San Diego, CA). After loading the amplified material onto independent flow cells, sequencing was carried out by running 36 cycles on the Illumina Genome Analyzer IIx at the BGI (Beijing, China). Illumina RNA-Seq quality scores converted to phred format (http://www.phrap.com/phred/) analyzed the quality of the RNA-Seq reads.

### Mapping to reference genome and annotated genes

*Colwellia psychrerythraea* 34H genome and gene information were available from NCBI (http://www.ncbi.nlm.nih.gov/genome/1067). After removing low quality reads and the reads containing adaptors, the remaining reads were aligned to the *C. psychrerythraea* 34H genome using software bowtie2 (version 2.0.2). Reads Per Kilobase per Million mapped reads (RPKM) were calculated for each transcript dividing number of mapped reads by the length of the transcripts and the number of total sequenced reads in this library.

### Quantification of *pfa* transcription in *C. psychrerythraea* by Quantitative real-time reverse transcription PCR (qRT-PCR)

After RNA isolation as described in the previous section, first-strand cDNA was synthesized using a PrimeScript RT reagent kit with gDNA eraser. Two microliters of RT product was used as a template for qRT-PCR with SYBR *PremixEx Taq* II kit. The primers used in this study are listed in Additional file 2: Table S2.The qRT-PCR mixture (25 μL) contained 2 μL of cDNA, 0.4 μM each gene-specific primer. The qRT-PCR was performed as follows: 4 min at 94 °C followed by 40 cycles of 30 s at 94 °C, 30 s at 56 °C, and 25 s at 72 °C, followed by a melting cycle from 56 °C to 94 °C to check for amplification specificity. To assess for DNA contamination, RNA samples were run without reverse transcriptase. The constitutively expressed 16S ribosomal RNA gene was used as an internal control for relative quantification, using the comparative threshold method.

## Results

### Optimal growth condition of *C. psychrerythraea*34H for DHA production

**Fig. 1:**
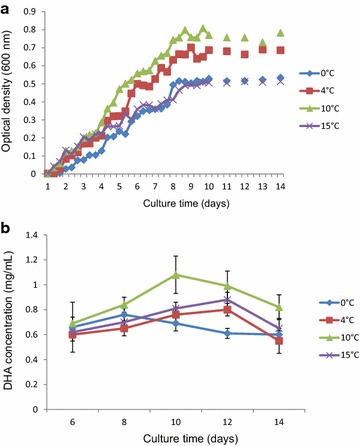
Growth curve of *C. psychreythraea* 34H under different temperatures (**a**). Effect of culturing time on DHA production in *C. psychreythraea* 34H (**b**)

*Colwellia psychrerythraea* 34H is a deep-sea bacteria containing a VLCPUFA Pfa synthase cluster according to its genomic sequence [15]. The 5 *pfaEABCD* genes responsible for *de novo* bacterial PUFA biosynthesis encode large, multi-domain protein complexes similar to type I iterative fatty acid and polyketide synthases [10]. PfaB has been described to be the chain length determinant in the VLCPUFA synthesis [23]. *Colwellia psychrerythraea* PfaB sequence showed 52 % identity with PfaB of DHA-producing *M. marina* and 27 % identity with PfaB of EPA-producing *S. baltica*, and was expected to allow the production of DHA [12]. In fact, the DHA production has been recently demonstrated [16]. In order to optimize the DHA production, we have tested different growth temperatures for *C. psychrerythraea* 34H. As shown in Fig. 1a, *C. psychrerythraea* 34H optimal growth temperature was found to be at 10 °C. Higher and lower growth temperatures resulted in longer generation times (Fig. 1a). DHA production by *C. psychrerythraea* 34H grown at various temperatures was also analyzed by GC. Maximum DHA production was observed at 10 °C for 10 days, with accumulation of 2.3 % DHA of the total fatty acids (Fig. 1b; Table 1). This DHA production was slightly lower than the production reported for *M. marina* (5.9 %). *M. marina* PfaA contains six ACP domains, while *C. psychrerythraea* only has five ACP domains. It was reported that the number of ACP domains was directly related to the capacity of VLPUFA synthesis [24].

**Table 1:** Effect of cerulenin (12 μg/mL) on the fatty acid composition of *C. psychrerythraea* 34H

Samples	Fatty acid composition (% of total peak area)															
	C10:0	C11:0	C12:0	C12:1^a^	C12:1d11	C13:0	C14:0	C14:1^b^	C14:1^c^	C14:1^d^	C15:0	C15:1	C16:0iso	C16:0	C16:1d9t	C16:1d7
	C16:1d9	C17:0	3OH-C12:0	C18:0	C18:1d9t	C18:1d9	C18:1d11	C19:0iso	C22:0	C22:1	C22:3n3	C22:6n3	C31:9			
M10	4.3 ± 0.2	0.4 ± 0.0	2.5 ± 0.1	0.3 ± 0.0	0.0 ± 0.0	0.2 ± 0.3	4.1 ± 0.0	0.3 ± 0.0	0.3 ± 0.0	0.0 ± 0.0	2.7 ± 0.0	2.9 ± 0.0	0.2 ± 0.1	28.3 ± 0.9	13.3 ± 0.2	9.0 ± 0.2
M10_C	17.6 ± 0.3	0.4 ± 0.0	5.9 ± 0.1	0.1 ± 0.1	0.0 ± 0.0	0.2 ± 0.3	26.7 ± 0.4	0.3 ± 0.0	0.3 ± 0.0	0.1 ± 0.2	1.1 ± 0.0	0.3 ± 0.2	0.0 ± 0.0	19.7 ± 0.2	0.6 ± 0.0	3.0 ± 0.1
M10	20.6 ± 0.3	0.7 ± 0.1	3.0 ± 0.1	0.7 ± 0.0	0.1 ± 0.1	2.6 ± 0.4	1.2 ± 0.2	0.1 ± 0.1	0.0 ± 0.0	0.0 ± 0.1	0.0 ± 0.0	2.3 ± 0.1	0.2 ± 0.0			
M10_C	8.0 ± 0.1	0.0 ± 0.0	3.7 ± 0.2	0.2 ± 0.2	0.0 ± 0.0	0.0 ± 0.0	0.0 ± 0.0	0.0 ± 0.0	0.3 ± 0.2	1.0 ± 0.1	0.1 ± 0.1	10.1 ± 0.1	0.3 ± 0.3			

This strain was cultured at 10 °C for 8 days with or without cerulenin. FAMEs were extracted from two samples and analyzed by GC or GC/MS (See “Methods”). The quantification of each fatty acid from two samples was repeated in triplicate (n = 3). The data was indicated as mean percentage of total peak area ± SEM

^a,b,c,d^ The double bonds of these fatty acids are still unknown. C16:0iso is branch fatty acid with 15 carbons in main chain, while C19:0iso is branch fatty acid with 18 carbon in main chain. These were confirmed by GC–MS

### Five genes involved in PKS pathway are essential to DHA formation

**Fig. 2:**
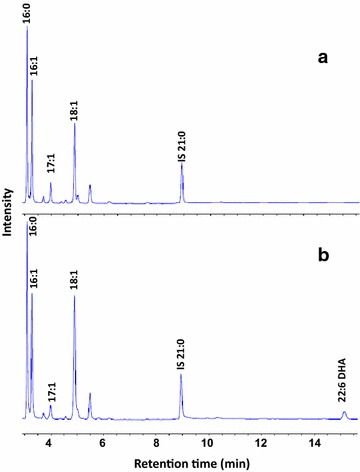
Confirmation of the functions of five *pfa* genes in *E. coli*. GC analysis of FAMEs from the *E. coli* harboring *pfaABCDE* (**b**) or empty vector (**a**)

Genes involved in PKS pathway including *pfaA*, *pfaB*, *pfaC*, *pfaD* (2′-nitropropane dioxygenase) and *pfaE* (4′-phosphopantetheinyl transferase), denoted as CPS_RS13885, CPS_RS13880, CPS_RS13875, CPS_RS13865 and CPS_RS13895 in the genome sequence, have high sequence similarity to corresponding *pfa* genes from other DHA-producing bacteria [10]. The function of these five genes was further confirmed by heterologous co-expression of them in *E. coli*. In the control *E. coli* transformant containing either of two empty vectors pColdI or pSTV28, 16:0, 16:1 and 18:1 were present as major components according to GC result. In contrast, an additional peak identified as DHA was detected in transformants harboring *pfaABCDE* from strain 34H. The DHA content was about 1.2 % of the total fatty acids (Fig. 2).

### Occurrence of multiple components of monounsaturated, polyunsaturated and hydroxyl fatty acids

**Fig. 3:**
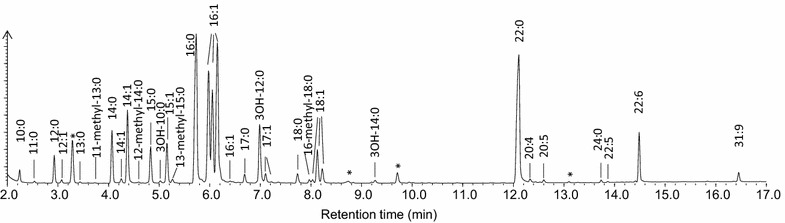
Gas chromatogram of *C. psychrerythraea* 34H total FAMEs. *indicated the unknown peaks

GC analysis of total fatty acid methyl esters (FAMEs) revealed the complexity of fatty acid profile in *C. psychrerythraea* 34H cells grown at 10 °C (Fig. 3). The major fatty acids were C16 fatty acids (saturate C16:0 and monosaturated C16:1), followed by C14 fatty acids and VLCPUFA DHA C22:6. Multiple monounsaturated C16 and C18 fatty acid peaks were also detected. These peaks were confirmed as C16:1^Δ9t^, C16:1^Δ7c^, C16:1^Δ9c^, C16:1^Δ11c^, C18:1^Δ9t^, C18:1^Δ7c^, C18:1^Δ9c^, C18:1^Δ11c^, both by GC analysis comparing to known standards, or by GC–MS analysis to confirm the mass spectra and determine the double bond position. Three major C16:1 peaks were detected, eluted at the corresponding retention time of FAMEs prepared from commercial available fatty acids C16:1^Δ9t^, C16:1^Δ6c^, C16:1^Δ9c^, with an additional shoulder peak that appeared in trace amount after C16:1^Δ9c^ FAME (Additional file 3: Figure S1). GC–MS analysis of these peaks confirmed all four peaks showed typical C16:1 FAME mass spectra, although with different retention times. While the presence of C16:1^Δ9t^ and C16:1^Δ9c^ was confirmed by GC–MS, the second C16:1 peak was identified as C16:1^Δ7^rather than C16:1^Δ6^ (Additional file 4: Figure S2). The tiny shoulder peak was identified as C16:1^Δ11c^. Similarly, three small C18:1 peaks were detected, and confirmed as C18:1^Δ9t^, C18:1^Δ9c^, C18:1^Δ11c^ by GC analysis comparing to the FAMEs prepared from commercial available C18:1^Δ9t^, C18:1^Δ9c^, C18:1^Δ11c^ fatty acid standards (Additional file 5: Figure S3) or by GC–MS (data not shown). Two hydroxyl fatty acids were also detected, being identified as 3-OH C10:0 and 3-OH C12:0 (Additional file 6: Figure S4). The position of the hydroxyl group was confirmed with TMS derivatives (Additional file 7: Figure S5) as described in “Methods” section. Trace amounts of branch fatty acids were also identified (Additional file 8: figure S6).

In addition to DHA, trace amounts of other VLCPUFA including C20:4ω3 and C20:5ω3 were also found (Additional file 9: Figure S7). The identities of these components were confirmed by GC analysis comparing the FAME standards, or by GC–MS analysis of FAMEs and/or their DMOX derivatives.

### Effect of cerulenin treatment on fatty acid composition of *C. psychrerythraea* 34H

**Fig. 4:**
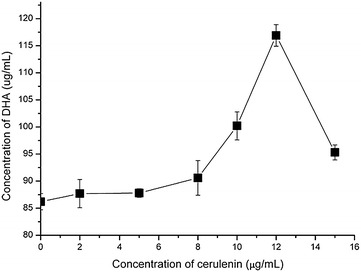
Effect of cerulenin on the production of DHA in *C. psychreythraea* 34H. *C. psychrerythraea*, cells were cultivated at 10 °C with shaking at 200 rpm for 24 h after cerulenin added

*Colwellia psychrerythraea* cells grown at 10 °C treated with the FAS inhibitor cerulenin resulted in a significant fatty acid profile change. The C16 and C18 monounsaturated fatty acids (MUFAs) especially C16:1^Δ9t^, C16:1^Δ7^, were greatly reduced after the treatment with cerulenin (Table 1). C16:1^Δ9t^ was decreased from 13.3 to 0.6 %, while C16:1^Δ7c^ decreased from 9.0 to 3.0 %. C16:1^Δ9c^ was decreased from 20.6 to 8.0 %. C18 MUFAs reduced from total of 2.7 % to undetectable level. In contrast, increased levels of short chain or medium chain saturated fatty acids (SFAs) C10:0, C12:0 and C14:0, hydroxyl fatty acids 3-OH C10:0 (trace amount) and 3-OH C12:0, as well as LCPUFA C20:4ω3, C20:5ω3 and C22:6ω3 were observed. At the optimal concentration of 12 μg/mL, cerulenin treatment resulted in up to 120 μg/mL of DHA (C22:6ω3) and 10.1 % to the total fatty acids (Fig. 4; Table 1).

### Transcriptome analysis of *C. psychrerythraea* after cerulenin treatment revealed the differential response of pathways

**Fig. 5:**
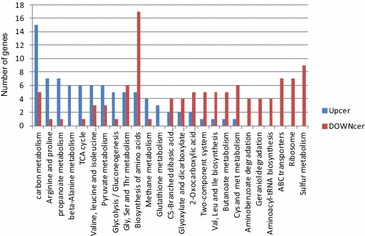
Summary of KEGG annotations for the number of genes. Distribution of up-regulated genes (Upcer) and down-regulated genes (DOWNcer) in the cerulenin treated sample

RNA-seq was used to analyze the expression patterns of *C. psychrerythraea* treated with or without 12 μg/mL of cerulenin. To compare the transcriptional activation or repression by cerulenin, normalized RPKM were calculated as described in materials and methods for each of the 5048 annotated *C. psychrerythraea* genes. As many as 240 genes were up-regulated by cerulenin treatment (twofold or greater, M10_C vs M10), while other 193 genes were down-regulated (twofold or greater, M10_C vs M10) (Additional file 10: Figure S8). For an overview of the metabolic changes that occurred after the cerulenin treatment we identified the KEGG pathways [25] corresponding to the up- or down-regulated genes. As shown in Fig. 5, catabolic pathways such as carbon metabolism, amino acid degradation pathways, TCA cycle or glucolysis pathways were up-regulated by cerulenin. On the other hand, amino acid biosynthesis, ribosomal genes or ABC transporters were down-regulated.

### Expression of most genes involved in PKS and fatty acid biosynthesis pathway was not affected by cerulenin

Despite the increased level of DHA under these conditions, supplementation of cerulenin did not increase the expression of the five *pfa* genes involved in PUFAs biosynthesis (Table 2). This result was further confirmed by qRT-PCR (Additional file 11: Table S3). Moreover, genes involved in FA biosynthesis were not obviously affected by supplementation of cerulenin (Table 2). RNA-seq analysis showed that the expression of *fabB* (CPS_RS17030), reported to be responsible for the elongation of unsaturated fatty acid and proven to be the target for cerulenin [18], was not inhibited at the mRNA level. Another gene CPS_RS00390 encoding for cis/tran isomerase that could alter the ratio of cis- to trans-esterified fatty acids in phospholipids [15] was not changed at RNA level, although the trans monounsaturated fatty acids (C16:1^Δ9t^ and C18:1^Δ9t^) were greatly reduced.

**Table 2:** Differential expression of genes involved in fatty acid metabolism in *C. psychrerythraea* without or with 12 μg/mL cerulenin at 10 °C

Locus ID	Gene ID	Gene description	Fold change (log_2_)
Gene involved in fatty acid biosynthesis			
CPS_RS06985	accA	acetyl-CoA carboxylase subunit A	0.28
CPS_RS04205	accB	acetyl-CoA carboxylase subunit B	0.47
CPS_RS04200	accC	acetyl-CoA carboxylase subunit C	0.39
CPS_RS17005	accD	acetyl-CoA carboxylase subunit D	−0.31
CPS_RS14645	fabA	3-hydroxydecanoyl-ACP dehydratase, isomerase	−0.21
CPS_RS17030	fabB	3-ketoacyl-ACP synthase I	0.96
CPS_RS10240	fabF	3-ketoacyl-ACP synthase II	0.41
CPS_RS09575	fabH	3-ketoacyl-ACP synthase III	0.38
CPS_RS10225	fabD	malonyl-CoA:ACPtransacylase	0.95
CPS_RS07145	fabG1	3-ketoacyl-ACP reductase	0.01
CPS_RS10230	fabG2	NADPH-dependent 3-ketoacyl-ACPreductase	−0.03
CPS_RS06960	fabZ	3-hydroxyacyl-ACP dehydratase	−0.06
CPS_RS01535	fabR	DNA-binding transcriptional repressor	−0.04
Gene involved in fatty acid degradation			
CPS_RS01760	fadA	3-ketoacyl-CoA thiolase	−0.57
CPS_RS01765	fadB	3-hydroxyacyl-CoA dehydrogenase	−0.92
CPS_RS05300	fadD1	Long chain fatty acid-CoA ligase	−1.51
CPS_RS15290	fadD2	Long chain fatty acid-CoA ligase	0.28
CPS_RS10085	fadE	Acyl-CoA dehydrogenase	0.40
CPS_RS04110	fadH	2,4-dienoyl-CoA reductase	0.55
CPS_RS15575	fadR	Fatty acid metabolism regulator	−0.39
Gene involved in fatty acid modification or lipid metabolism			
CPS_RS00390	cti	fatty acid cis/trans isomerase	0.11
CPS_RS10220	plsX	glycerol-3-phosphate acyltransferase	−0.30
CPS_RS19415	plsY	glycerol-3-phosphate acyltransferase	0.31
CPS_RS00605	GPAT	glycerol-3-phosphate acyltransferase	0.21
CPS_RS04525	LPAAT	1-acyl-sn-glycerol-3-phosphate acyltransferase	0.06
CPS_RS21310	LPAAT	1-acyl-sn-glycerol-3-phosphate acyltransferase	−0.08
CPS_RS04435		acyltransferase	0.02
CPS_RS08065		acyltransferase	0.25
CPS_RS14560		acyltransferase	0.55
CPS_RS21745		acyltransferase	0.52
CPS_RS22505		acyltransferase	0.70
CPS_RS22520		acyltransferase	−0.20
Genes involved in PKS pathway			
CPS_RS13885	pfaA	polyunsaturated fatty acid synthase	−0.58
CPS_RS13880	pfaB	polyunsaturated fatty acid synthase	−0.37
CPS_RS13875	pfaC	polyunsaturated fatty acid synthase	0.03
CPS_RS13865	pfaD	polyunsaturated fatty acid synthase	0.60
CPS_RS13895	pfaE	4′-phosphopantetheinyl transferase	−0.51

Fold change (log_2_ values) in transcript levels under specified conditions as determined by RNA-seq. Transcript abundance obtained from RNA-seq data is indicated as RPKMs (See “Methods” section)

However, several genes involved in fatty acid degradation, such as *fadD1* (CPS_RS05300) and *fadB* (CPS_RS01765), were down-regulated. Besides, the genes encoding for acyltransferases involved in polar lipid and neutral lipid formation, including glycerol-3-phosphate acyltransferase (CPS_RS10220, CPS_RS19415, CPS_RS00605), 1-acyl-sn-glycerol-3-phosphate acyltransferase (CPS_RS04525, CPS_RS21310) were not affected. Three out of six genes for other annotated acyltransferases (CPS_RS04435, CPS_RS08065, CPS_RS14560, CPS_RS21745, CPS_RS22505 and CPS_RS22520) had slight increased expression level. Their functions are to be further investigated.

### Top up-regulated genes by cerulenin

**Fig. 6:**
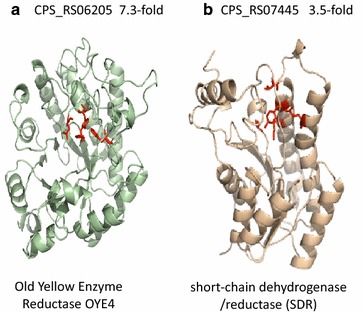
Structural prediction analysis of the CPS_RS06205 (**a**) and CPS_RS07445 (**b**) revealed their similarity to old yellow enzyme reductase OYE4 and short chain dehydrogenase/reductase, respectively

The most up-regulated genes in the cerulenin-treated cells were found to be the operons containing oxidoreductase genes. CPS_RS06205 gene, coding for an oxidoreductase, FAD/FMN-binding protein was dramatically upregulated (log_2_ fold change was 7.31) (Additional file 12: Table S4). We carried out the structural prediction and modeling analysis of CPS_RS06205 using the Protein Homology/analogY Recognition Engine (PHYRE) Web server [26]. As shown in Fig. 6a, CPS_RS06205 was found to be homolog to the Old Yellow Enzymes (OYEs, NCBI accession number AAN56390), flavin-dependent enoate reductases (EC 1.6.99.1) that catalyze the stereoselective hydrogenation of activated double bonds of different alkenes [27]. Another highly up-regulated gene by cerulenin was CPS_RS04135. CPS_RS04135 belongs to the crotonyl-CoA carboxylase-reductase (CCR) family, involved in extender-unit biosynthesis of polyketides [28]. Moreover, CPS_RS07445, upregulated 11-fold, codes for a short chain dehydrogenase/reductase family oxidoreductase (Fig. 6b). CPS_RS07440, upregulated sixfold, codes for a FMN-dependent NADH-azoreductase. Genes CPS_RS18085 (11-fold) and CPS_RS18095 (fourfold), also code for allantoate amidohydrolase and NADPH-dependent F420 reductase, respectively (Additional file 12: Table S4).

## Discussion

### Complexity of *C. psychrerythraea* fatty acid profile

Although the major fatty acids in *C. psychrerythraea* 34H cells were C16 fatty acids, the very complicated fatty acid profile was uncovered in this work. These FAs included short and medium chain SFAs, odd number carbon length FAs, short and medium chain MUFAs, multiple component of monounsaturated C16:1 or C18:1, branch fatty acids, PUFAs and 3-HFAs, although some of the components had very low levels. The existence of these components was confirmed by GC–MS analysis. The occurrence of trans monounsaturated C16:1 and PUFAs in 34H has also been recently reported [16]. Under our experimental conditions, we detected more components that were not reported previously. These included trace amounts of C11:0, C13:0, three isomers of C14:1, 3-OH C10:0, C18:1^Δ9trans^, and branch fatty acids C16:0iso and C19:0iso. Such fatty acid profile could indicate the complexity of fatty acid synthesis and modification pathways in strain 34H.

We showed that the fatty acid profile was differentially affected by cerulenin treatment. Cerulenin, a specific inhibitor of condensing enzymes, had been reported to inhibit MUFA synthesis but not PUFA synthesis in deep-sea bacteria *M. marina* [19]. This is consistent to our result that C16:1 and C18:1 MUFAs were significantly decreased, especially the trans fatty acids, while the VLCPUFA DHA increased. We also showed the enhanced levels of C10:0, C12:0, C14:0 and 3-hydroxy C12:0 after cerulenin treatment (Table 1). The condensing enzymes principally extend the fatty acid chain length by two carbons in each fatty acid synthesis cycle. Inhibition of the condensing enzymes by cerulenin could explain the accumulation of shorter chain length fatty acid, and decreased the longer chain length fatty acids. Indeed, C16:0 and C18:0 FAs were both reduced after cerulenin treatment. However, the significant increase of C14:0 levels might suggest the condensing step from C12:0 to C14:0 was not inhibited, same as the step from C8:0 to C10:0. It is possible that cerulenin might inhibit these steps differently, or these steps might be catalyzed by different condensing enzyme isomers. In addition, C16 and C18 MUFAs were decreased. *Escherichia coli* possesses at least three condensing enzymes: FabH selectively uses acetyl-CoA to initiate the fatty acid synthesis pathway, and FabB/FabF carry out the subsequent rounds of fatty acid elongation which condense malonyl-ACP with acyl-ACP to extend the acyl chain by two carbons [29]. FabF is confirmed to be the sole condensing enzyme for subsequent elongation step in this strain [30]. In contrast, *Bacillus subtilis* contains two FabH isozymes (FabHA and FabHB) which differ from *E. coli* FabH in that they could initiate both the straight and branched chain fatty acid synthesis. Similar to *E. coli*, only one initial condensing enzyme FabH (CPS_RS09575) and two other condensing enzymes, FabB (CPS_RS17030) and FabF (CPS_RS10240), were denoted in the genome of 34H.

### No effect of PKS pathway at transcriptional level

In order to better understand the effect of cerulenin on the fatty acid level change, we performed the transcriptome analysis. To our surprise, there was no obvious change of PUFA PKS pathway at the transcription level after cerulenin treatment. The five *pfa* genes were confirmed to be essential to DHA production in strain 34H by heterologous co-expression in *E. coli*. However, all these five genes did not increase the expression level after cerulenin treatment, although the DHA level was significantly increased. This might suggest the increase of DHA content in *C. psychrerythraea* was not regulated by *pfa* gene cluster at the transcriptional level when cells were treated with cerulenin. It was reported that cultivation of *P. profundum* strain SS9 at elevated hydrostatic pressure or reduced temperature did not increase *pfa* gene expression despite the resulting increase in percentage composition of EPA under these conditions [31]. We proposed that there could be some other genes or transcriptional factors related to the increase of DHA accumulation. Another explanation could be that the availability of malonyl-CoA for PKS pathway is higher after cerulenin treatment.

Besides, *pfa* genes are regulated by PfaR. PfaR is predicted to be an inactive transcriptase that does not seem to be affected for any effectors. Therefore, it has sense that cerulenin affecting fatty acid profile does not affect PfaR binding and thus does not affect *PfaABCD* transcription. A BLAST search against current databases using the sequence of PfaR revealed more than 100 putative PfaR homologs in marine bacteria, but not in *M. marina* (data not shown). In fact the *PfaRABCD* cluster is conserved in all these marine bacteria. Moreover, we carried out structural prediction and modeling of PfaR using the PHYRE. 263 residues (91 %) of PfaR could be modeled at >90 % accuracy using structure 4FCY (crystal structure of the bacteriophage mu transpososome) as a model, although the sequence identity between PfaR and 4FCY is only 14 % (unpublished data). MuA, like many other DNA transposases, and retroviral integrases share a conserved RNaseH-like or “DDE” catalytic domain, named for the 3 Mg^2+^ binding carboxylates in their active sites. However, these catalytic residues are not conserved in PfaR. Thus, we think PfaR is not an active transposase but rather a transcriptional regulator.

Similarly, the gene CPS_RS00390 coding for a cis/trans isomerase that could alter the ratio of cis- to trans-esterified fatty acids in phospholipids was not obviously changed at RNA level, although the trans monounsaturated fatty acids (C16:1^Δ9t^ and C18:1^Δ9t^) were greatly reduced. Moreover, considering that the reduction level of C16:1^Δ9t^ was much higher than that of C16:1^Δ9c^, a more complicated effect could exist rather than a simple effect of CPS_RS00390 expression level.

### Down-regulation of some genes involved in fatty acids degradation

Bacterial FASII system contained basically the same set of enzymatic domains as PKS [30]. Although FAS and PKS used the same substrates, acetyl-CoA and manoly-CoA, the final products were quite distinctive. In general, FAS was responsible for the essential short and medium chain fatty acids while PKS was only capable of production of VLCPUFAs [32]. Until now, the mechanism of cross talk between FAS and PKS was still unknown. FAS pathway can be classified into fatty acids biosynthesis pathway and fatty acids degradation pathway. As shown in Table 2, most of the genes involved in the fatty acid biosynthesis pathway were not obviously affected in the presence of cerulenin. Notably, one of those genes, *fabB* encoding for 3-ketoacyl-ACP synthase, was responsible for the elongation of unsaturated fatty acids. FabB activity was demonstrated to be irreversibly inhibited by cerulenin [18]. Although the content of MUFAs, such as C16:1^Δ9t^, C16:1^Δ7c^ and C16:1^Δ9c^ was indeed decreased (Table 1), the transcriptional expression of *fabB* was not down-regulated in the presence of cerulenin in this test, but slightly up-regulated (Table 2). It was also demonstrated that transcription of the operon containing the *fabF* gene from *Bacillus subtilis* increased eightfold in response to cerulenin treatment [33]. We speculated that the minor increase of *fabB* or *fabF* might be to compensate the low activity of FabB/FabF protein.

In contrast, two genes (*fadB* and *fadD1*) involved in the fatty acid degradation pathway were dramatically down-regulated (Table 2). In the first step of long chain fatty acid degradation, fatty acids would be transported across the cell membrane via a transport and acyl-activation mechanism. Gene *fadD* encoding an inner membrane-bound acyl-CoA synthase was involved in this process. In general, there is only one acyl-CoA synthase in *E. coli* with broad substrate specificity [30]. However, in *C. psychrerythraea*, two genes *fadD1* and *fadD2*, were both denoted as acyl-CoA synthases. Only *fadD1* was dramatically down-regulated while *fadD2* was slightly up-regulated (Table 2). Therefore, two FadD enzymes might have different substrate specificities based on the changed fatty acids profile. This result awaits further investigation. The other down-regulated gene *fadB* was thought to encode for enoyl-CoA hydratase/3-hydroxyacyl-CoA dehydrogenase. This enzyme converted enoyl-CoA to 3-ketoayl-CoA via 3-hydroxylacyl-CoA through hydration and oxidation [30]. Both *fadD* and *fadB* played critical roles in fatty acid degradation.

The final degradation of acyl-CoA is achieved by β-oxidation pathway. In *E. coli*, gene *fadE* encoding for acyl-CoA dehydrogenase, was demonstrated to be responsible for the conversion of acyl-CoA to enoyl-CoA. This gene was also thought to be the only acyl-CoA dehydrogenase gene in *E. coli* [34]. According to the genome sequence of strain 34H, CPS_RS10085, denoted as *fadE*, codes for an acyl-CoA dehydrogenase. However, there were six other genes denoted as acyl-CoA dehydrogenase as well in this strain (Additional file 13: Figure S9). Notably, only CPS_RS02980 and CPS_RS10085 were up-regulated while the others including CPS_RS10075, CPS_RS05245, CPS_RS12125, CPS_RS20065 and CPS_RS02940 were down-regulated (Additional file 13: Figure S9). The expansion of acyl-CoA dehydrogenase gene family might be important to the selective oxidation of short (C4–C6), medium (C8–C14), long (C16–C18) and very long (>C20) chain fatty acids. This could be one of the reasons led to the higher accumulation of DHA after cerulenin treatment.

### Other top regulated genes by cerulenin might contribute to the changes of fatty acid profile in strain 34H

Except for fatty acid metabolism related genes, other significantly regulated genes were also investigated. Three of the top 20 up-regulated genes are TetR family regulators (CPS_RS06200, CPS_RS07325 and CPS_RS07450, Additional file 12: Table S4). They belong to one-component systems in which a single polypeptide contains both a sensory domain and a DHA-binding domain [35]. They are widely associated with many biological processes, such as antibiotic resistance and fatty acids synthesis [36, 37]. These proteins have several homologs in the Protein Data Bank (PDB). One of them is FadR. FadR binds one lauroyl (C12)-CoA molecule per FadR monomer, with its acyl chain moiety in the center of the FadR molecule, enclosed within a tunnel-like substrate-binding pocket surrounded by hydrophobic residues, and the CoA moiety interacting with basic residues on the protein surface [36]. Recently, *fadR* was demonstrated to be an important transcriptional factor that was capable of positively control the *fabA* and *fabB/F* genes in *E. coli* [37]. Over-expression of *E. coli FadR* could dramatically increase the fatty acid titer by 7.5-fold over wild-type strains. Moreover, the unsaturated fatty acids including 14:1, 16:1 and 18:1 were increased [37]. Besides, FadR was demonstrated to dissociate with C16–C18 fatty acids but not with C10 fatty acids [38]. Thus, cerulenin is not expected to affect FadR regulation.

The other most up-regulated gene was CPS_RS06205. The predicted protein structure of this gene showed high similarity to OYEs. OYEs have been found to be highly expressed in the EPA producing strain *S. oneidensis* [39]. The expression of OYE in *S. oneidensis* was observed in the absence of cerulenin. Thus, CPS_RS06205 upregulation seems to be related to the metabolic changes that drive DHA accumulation and not to be the cerulenin effect itself.

CPS_RS04135 denoted as a CCR family gene, was dramatically up-regulated as well. It could reduce double bond in the accumulated MUFAs and other fatty acids to produce the extender units needed by PKS for DHA synthesis. Therefore, the accumulation of precursors might be one possible reason for DHA increase in cerulenin-treated cells. On the other side, CPS_RS07330 (14-fold) is homolog to the multidrug efflux protein AcrB. AcrA/B system is controlled by the TetR-like protein ArcR. This efflux system could be upregulated to cope with the toxicity of cerulenin. The ability to reduce the dye 2,3,5-triphenyltetrazolium chloride (TTC) has been shown to be directly associated with EPA production in marine Gram-negative bacteria in solid or liquid medium [40]. The up-regulated oxidoreductases could be responsible of this reduction. Moreover, detoxification of H_2_O_2_ is other characteristic of PUFA producing deep-sea bacteria [41] that could also be due to the expression of different reductases.

Although *C. psychrerythraea* 34H cell produces DHA, it can only grow at relatively low temperature, and the transformation system for genetic modification has not been set up. However, this strain synthesizes DHA via PKS pathway. Compared to conventional desaturase/elongase pathway, PKS is an anaerobic pathway and less NADPH was needed for the whole process. More important, unlike desaturase/elongase pathway, the final products of PKS are only VLCPUFAs. Our current work is to seek and investigate good gene candidates from microbes harboring PKS pathway. These genes can be engineered into other expression systems such as oleaginous yeast *Y. lipolytica* [4] to produce high levels of VLCPUFAs.

## Conclusions

In summary, the complexity of psychrophilic *C. psychrerythraea* fatty acid profile was revealed by GC or GC–MS. Cerulenin treatment of this strain resulted in enhanced accumulation of short and medium chain saturated fatty acids, 3-hydroxy fatty acids, and DHA, at the expense of C16 and C18 saturated and MUFAs. Five *pfa* genes involved in PKS pathway were demonstrated to be responsible for DHA production by heterogeneous expression in *E. coli*. However, genes involved in polyketide synthase pathway and fatty acid biosynthesis pathway were not obviously affected by cerulenin treatment. In contrast, several genes involved in fatty acid degradation or β-oxidation pathway were dramatically reduced at the transcriptional level. The results suggested that acyl-CoA dehydrogenase gene family involved in the first step of β-oxidation pathway may be important to the selectivity of degraded fatty acids. In addition, inhibition of FabB protein by cerulenin may lead to the accumulation of malonyl-CoA, which is the substrate for DHA formation.

## Additional files

10.1186/s12934-016-0431-9 Sequences of primers used for vector construction.
10.1186/s12934-016-0431-9 Sequences of primers used for qRT-PCR.
10.1186/s12934-016-0431-9 Partial gas chromatogram of *C. psychrerythraea* 34H. A. FAMEs prepared from standards C16:1^Δ9t^, C16:1^Δ6c^, C16:1^Δ9c^ in an approximated ration of 7:4:10. B. Three major C16:1 peaks eluted at the corresponding retention time of C16:1^Δ9t^, C16:1^Δ6c^, C16:1^Δ9c^ standards, with shoulder peak appearing after C16:1^Δ9c^. GC–MS analysis confirmed these peaks were C16:1^Δ9t^, C16:1^Δ7c^, C16:1^Δ9c^, C16:1^Δ11c^.
10.1186/s12934-016-0431-9 Mass spectra of four C16:1 peaks in order, at retention time of 5.975, 6.050, 6.150 and 6.275 min.
10.1186/s12934-016-0431-9 Partial gas chromatogram of *C. psychrerythraea* 34H. A. FAMEs prepared from standards C18:1^Δ9t^, C18:1^Δ6c^ in an approximated ration of 1:10. B. Four C18:1 peaks eluted at the corresponding retention time of C18:1^Δ9t^, C18:1^Δ6c^, C18:1^Δ9c^, C18:1^Δ11c^ standards. GC–MS analysis confirmed these peaks were C18:1^Δ9t^, C18:1^Δ7c^, C18:1^Δ9c^, C18:1^Δ11c^.
10.1186/s12934-016-0431-9 Mass spectra of 3-hydroxyl fatty acid methyl esters. A. 3OH-C10:0 FAME at 5.025 min (M + 202); B. 3OH-C12:0 FAME at 6.992 min (M + 230); C. 3OH-C14:0 FAME at 9.275 min (M + 258).
10.1186/s12934-016-0431-9 Mass spectra of TMS derivative hydroxy fatty acid methyl esters and their TMS derivatives. A. 3OH-C12:0 FAME (M + 302); B. 3OH-C14:0 FAME (M + 330).
10.1186/s12934-016-0431-9 Mass spectra of branch chain fatty acid methyl esters. A. C14:0 FAME at 4.075 min (M + 242); B. 12-methyl-C14:0 FAME at 4.600 min (M + 256); C. C15:0 FAME at 4.833 min (M + 256). The different ratio of mass ion between m/z 199 and 213 in B and C suggested B could be the anteiso isomer of C15:0 FAME, i.e., 13-methyl-C14:0.
10.1186/s12934-016-0431-9 Mass spectra of polyunsaturated fatty acid methyl esters C20:4, C20:5, and C22:5.
10.1186/s12934-016-0431-9 Statistical analysis of differential expressed genes after cerulenin treatment.
10.1186/s12934-016-0431-9 Analysis of *C. psychrerythraea pfa* gene expression by RNA-seq and qRT-PCR.
10.1186/s12934-016-0431-9 Differentially expressed genes in *C. psychrerythraea* treatment without or with 12 μg/mL cerulenin at 10 °C.
10.1186/s12934-016-0431-9 Selective down-regulation of oxidation pathway genes. Bold indicated the gene had higher expression level in cerulenin-treated sample (M10_C) than in untreated sample (M10).
