# Supplementary material for: Effect of cerulenin on fatty acid composition and gene expression pattern of DHA-producing strain Colwellia psychrerythraea strain 34H
Source: Microb Cell Fact. 2016 Feb 6;15:30. doi: 10.1186/s12934-016-0431-9 (PMC4744452; doi:10.1186/s12934-016-0431-9)
Supplement: Supplementary file 1 — 10.1186/s12934-016-0431-9 Sequences of primers used for vector construction. [file 12934_2016_431_MOESM1_ESM.docx]

**Additional file 1: Table 1** Sequences of primers used for vector construction

| Primer name | Nucleotide sequence (5’-3’) |
| --- | --- |
| mpfaA-F | CG GAATTCATGGTAAATATTAGACGGATGATAATGAGC |
| mpfaD-R | ACGC GTCGACCTAATCTTCGCTACGATAGCCAGCCAG |
| mpfaE-F | CG GGTACCATGACTTCTTTTTCTCAATCTGAACT |
| mpfaE-R | CG GGATCC TTAGATTTCCTGATAACCAAGTAGG |
