# Supplementary material for: Effect of cerulenin on fatty acid composition and gene expression pattern of DHA-producing strain Colwellia psychrerythraea strain 34H
Source: Microb Cell Fact. 2016 Feb 6;15:30. doi: 10.1186/s12934-016-0431-9 (PMC4744452; doi:10.1186/s12934-016-0431-9)
Supplement: Supplementary file 2 — 10.1186/s12934-016-0431-9 Sequences of primers used for qRT-PCR. [file 12934_2016_431_MOESM2_ESM.docx]

**Additional file 2: Table S2** Sequences of primers used for qRT-PCR

| Primer name | Nucleotide sequence (5’-3’) |
| --- | --- |
| 16srRNA-F | CCTACGGGAGGCAGCAG |
| 16srRNA-R | ATTACCGCGGCTGCTGG |
| 3105-F | cagcgctgtagaaccaacaa |
| 3105-R | ccaacctgacgtagggtcat |
| pfaA-F | CGCAGATGGAACTGTTGCTA |
| pfaA-R | TAACTCGCCTGCTTCTTGGT |
| pfaB-F | GCCGATTGTCTACGCGTTAT |
| pfaB-R | AGCAATCGCTTTGCTCATTT |
| pfaC-F | TCAAAACCGCGTACCTAACC |
| pfaC-R | CTCACCTTGCACTTCGACAA |
| pfaD-F | GTTTGAAATGGGCGTGAAGT |
| pfaD-R | TACCTGCCCAAACATCATCA |
| pfaE-F | aaccacaaatttggcgtagc |
| pfaE-R | ccaagtaggggcagggtatt |
| 3975F2 | GGGCGTGGTCAGTTTTTAGA |
| 3975R2 | TCGCGTTGTAATTCAAACCA |
| 3379F1 | GCAACAGCGACCGAATTTAT |
| 3379R1 | CCCTGAGAGAACGAGACAGG |
| 3380F1 | AGCGCTCAACCTTTTGTTGT |
| 3380R1 | ACCATGTTTGTCAGCCAGTG |
| 3164F2 | GCCCTAGTGCCAAGTACTGC |
| 3164R2 | AGTGTCGCCACTTCGATTTT |
| 4268F1 | AAAACTCGGCTAACGCAGAA |
| 4268R1 | CATGCCATTTTTGTCGATGT |
| 2094F1 | TTGAAGCGGCATTAAAATCA |
| 2094R1 | ACGCAAAAATGGAACCTCAG |
| 1335F2 | CGGAATTTGCAGGTTATGCT |
| 1335R2 | CCAGATAAGAAACGCCCAAA |
| 3161F1 | CGCCTACTCTTCCTGGTCAA |
| 3161R1 | GGGGCAATTGGGAAATTACT |
| 1314F1 | CAGAAGCACTTCCTTACCTTGA |
| 1314R1 | TCCGAGATAAACCGACGAAG |
| 3994F2 | TGAAGTATCGGCAGCACTTG |
| 3994R2 | TAACGGTTTTGCCATCAACA |
| 3984F2 | ACAAGGCGTGGAAGAACAAC |
| 3984R2 | AGCACAACACGACGGTCATA |
| 1411F1 | CGCACCAACCATTGATATTG |
| 1411R1 | AGAGATGCCCTCTACCGTGA |
| 0931F1 | CTAGCCCAAAACAGGGTGAA |
| 0931R1 | ATCGGCGTAAGATTTTGCAT |
| 1210F2 | AGCCTGGATCAGCATCAGTT |
| 1210R2 | GGTAAAGCTCGCACTCCAGA |
| 1608F1 | GCCTCTAAAGCGGCACTAAA |
| 1608R1 | TATGTTGGGCCTGGGTTTAC |
| 1654F1 | AATGGACCAGTTAGGCGTTG |
| 1654R1 | TTGCCCAAAAATCTTCAAGG |
| 1653F1 | CAATTGCATCCGATGAACAA |
| 1653R1 | GGCAAATTCAGAGCCCATAA |
| 1396F1 | AACCGCAGAGCAGGATATTG |
| 1396R1 | CGCTGATGCCTTTTTCTTCT |
| 1652F1 | AAGTTGGCGCTTTGGTCTTA |
| 1652R1 | GACCGACTTGCCCACTGTAT |
| 1398F2 | CATGGGGACCACTGAAAATC |
| 1398R2 | CACAAGGCAATCCCTGTTTT |
| 1399F1 | TTGATGTCGGTATTTATTAACTGG |
| 1399R1 | TCGCCTATCGTCGGTAGAAT |
| 1397F2 | GCGGGCTTAATCATTTCTGA |
| 1397R2 | TCCAGCCTTCAATTTGTGCT |
| betB1F2 | GTGCTGGTGAAGTGGGTTCT |
| betB1R2 | AGCACCTGCCATGACTTTCT |
