# Supplementary figures and images for: Effect of cerulenin on fatty acid composition and gene expression pattern of DHA-producing strain Colwellia psychrerythraea strain 34H
Source: Microb Cell Fact. 2016 Feb 6;15:30. doi: 10.1186/s12934-016-0431-9 (PMC4744452; doi:10.1186/s12934-016-0431-9)

## Slide 1
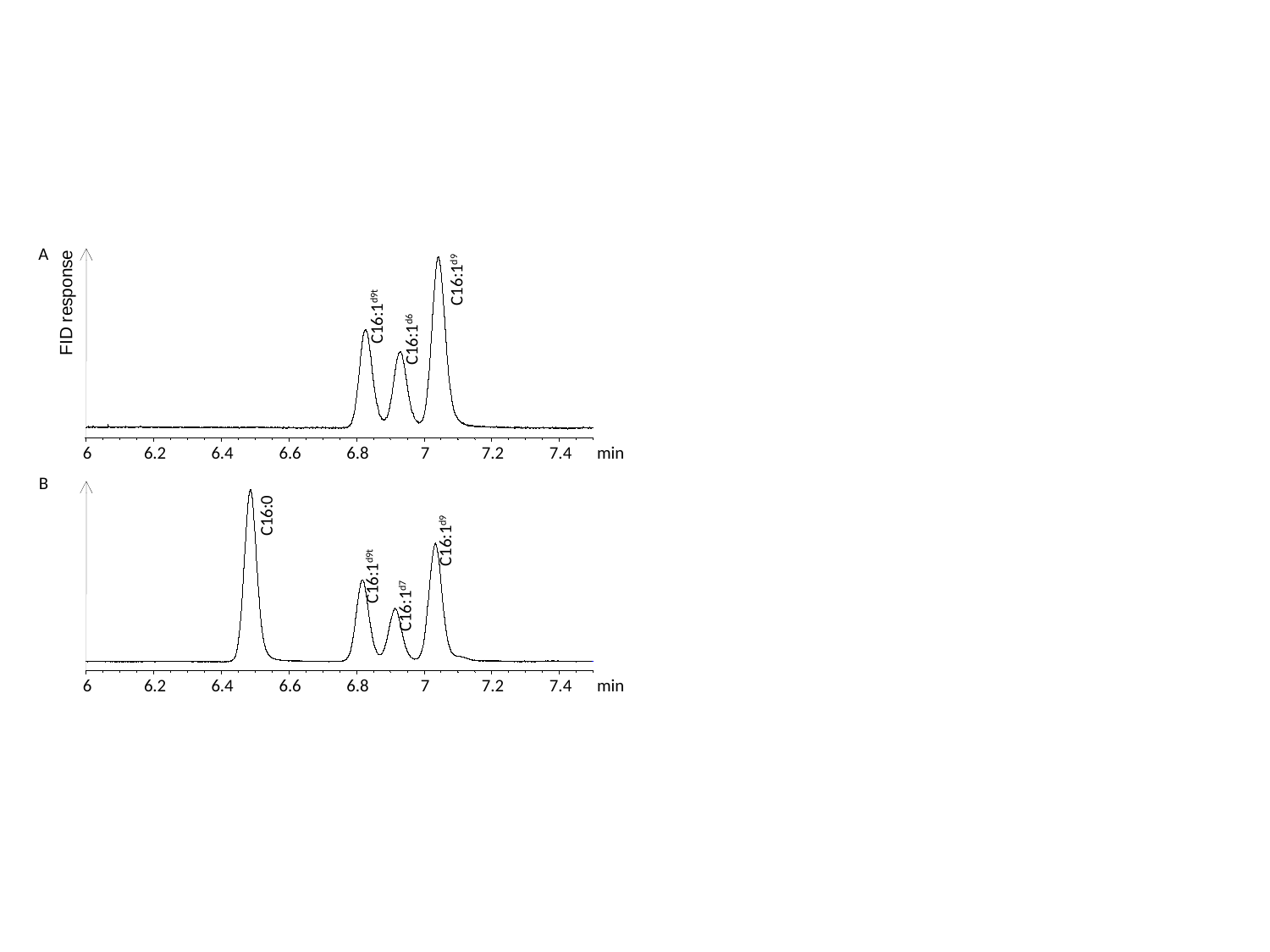

A
 C16:1d9
 C16:1d9t
 C16:1d6
6
6.2
6.4
6.6
6.8
7
7.2
7.4
min
FID response
B
 C16:0
 C16:1d9
 C16:1d9t
 C16:1d7
6
6.2
6.4
6.6
6.8
7
7.2
7.4
min

Supplement: Supplementary file 3 — 10.1186/s12934-016-0431-9 Partial gas chromatogram of C. psychrerythraea 34H. A. FAMEs prepared from standards C16:1Δ9t, C16:1Δ6c, C16:1Δ9c in an approximated ration of 7:4:10. B. Three major C16:1 peaks eluted at the corresponding retention time of C16:1Δ9t, C16:1Δ6c, C16:1Δ9c standards, with shoulder peak appearing after C16:1Δ9c. GC–MS analysis confirmed these peaks were C16:1Δ9t, C16:1Δ7c, C16:1Δ9c, C16:1Δ11c. [file 12934_2016_431_MOESM3_ESM.pptx]

## Slide 1
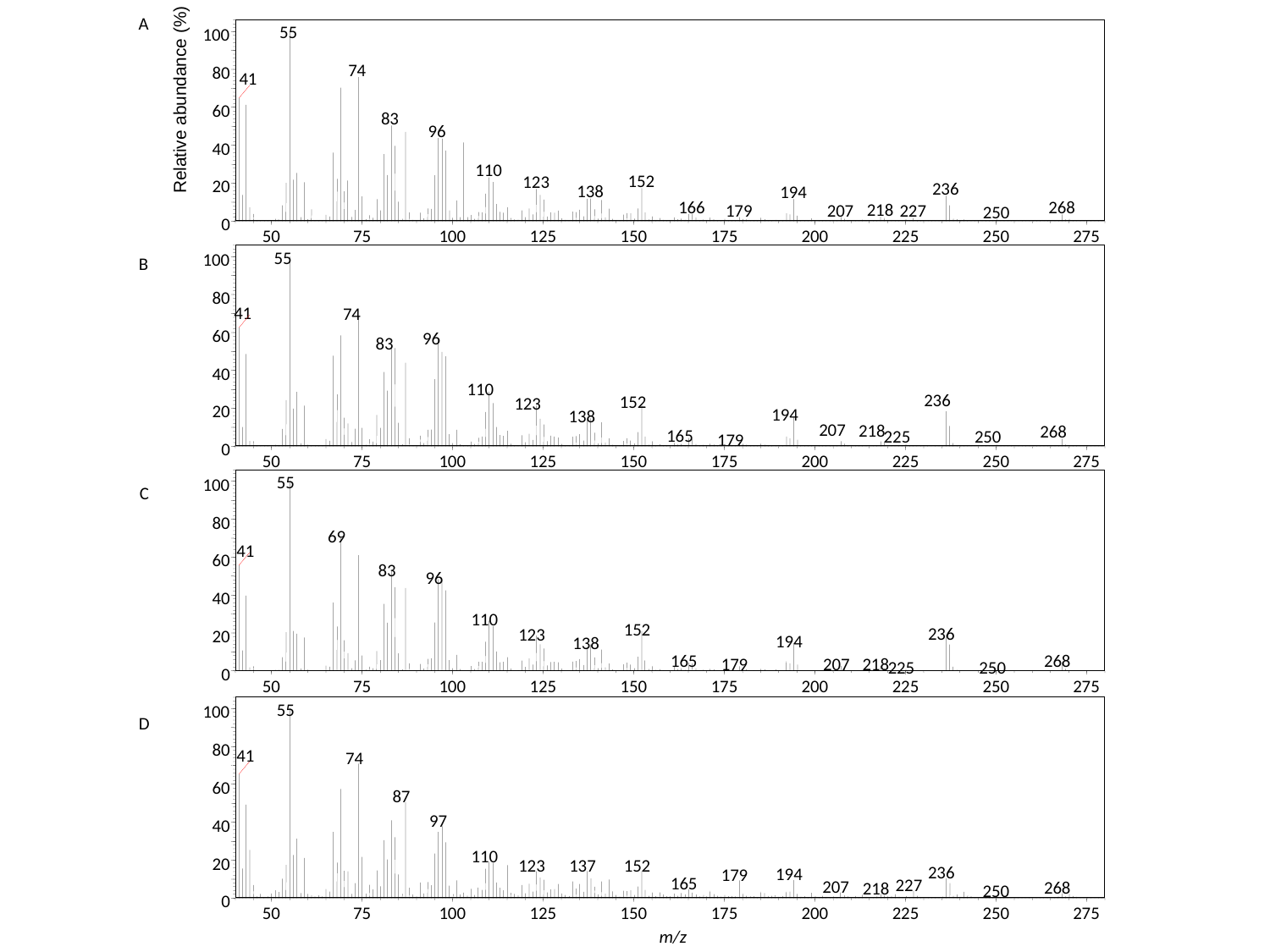

Relative abundance (%)
A
55
100
74
80
41
60
83
96
40
110
152
123
20
236
138
194
166
268
218
179
207
227
250
0
50
75
100
125
150
175
200
225
250
275
55
100
80
41
74
60
96
83
40
110
236
152
123
20
194
138
207
218
268
165
225
250
179
0
50
75
100
125
150
175
200
225
250
275
B
55
100
80
69
41
60
83
96
40
110
152
236
123
20
194
138
268
165
207
218
179
225
250
0
50
75
100
125
150
175
200
225
250
275
C
55
100
80
41
74
60
87
97
40
110
20
137
152
123
236
194
179
165
227
207
268
218
250
0
275
50
75
100
125
150
175
200
225
250
D
m/z

Supplement: Supplementary file 4 — 10.1186/s12934-016-0431-9 Mass spectra of four C16:1 peaks in order, at retention time of 5.975, 6.050, 6.150 and 6.275 min. [file 12934_2016_431_MOESM4_ESM.pptx]

## Slide 1
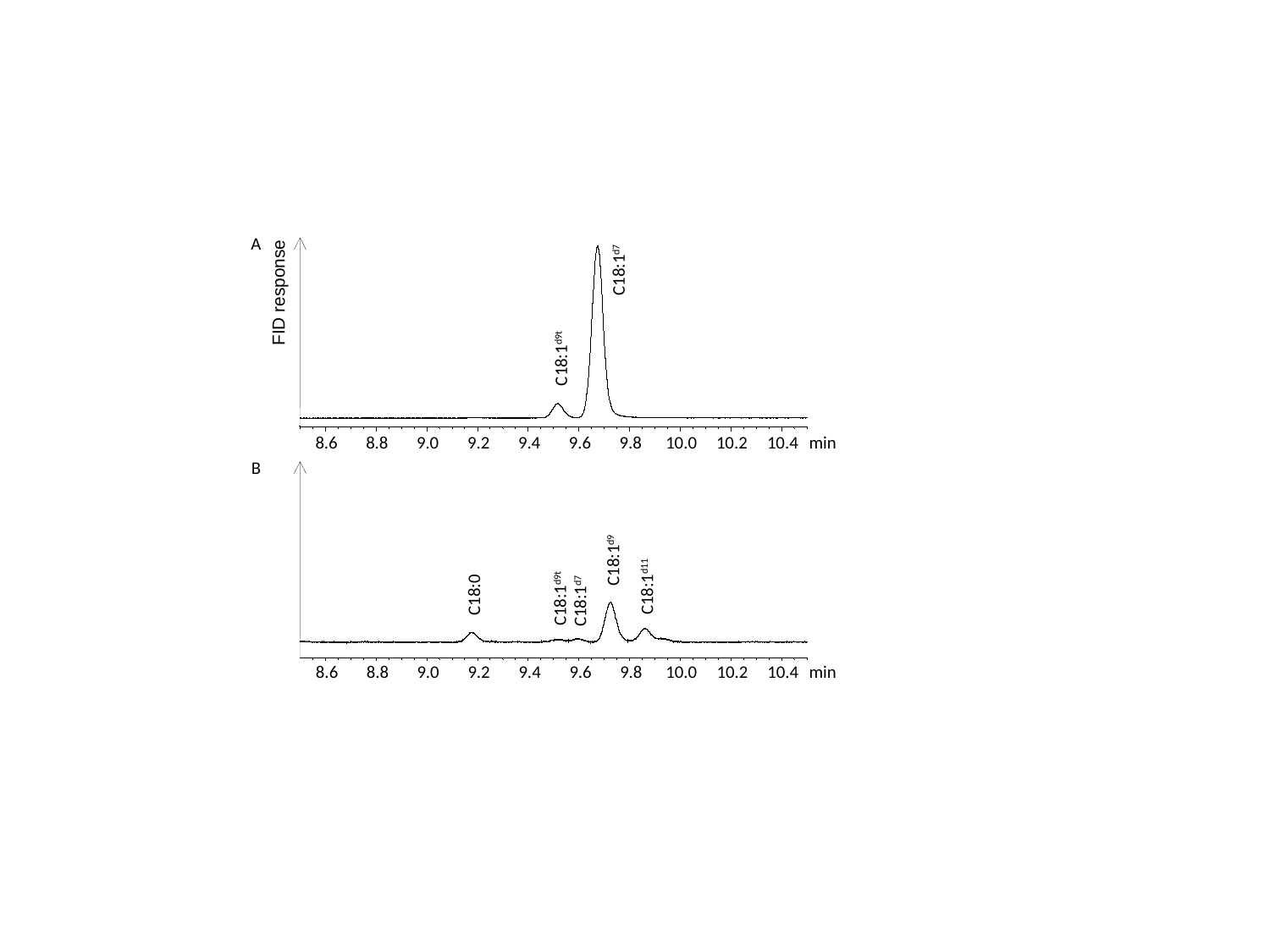

A
FID response
 C18:1d7
 C18:1d9t
8.6
8.8
9.0
9.2
9.4
9.6
9.8
10.0
10.2
10.4
min
B
 C18:1d9
 C18:1d11
 C18:0
 C18:1d9t
 C18:1d7
8.6
8.8
9.0
9.2
9.4
9.6
9.8
10.0
10.2
10.4
min

Supplement: Supplementary file 5 — 10.1186/s12934-016-0431-9 Partial gas chromatogram of C. psychrerythraea 34H. A. FAMEs prepared from standards C18:1Δ9t, C18:1Δ6c in an approximated ration of 1:10. B. Four C18:1 peaks eluted at the corresponding retention time of C18:1Δ9t, C18:1Δ6c, C18:1Δ9c, C18:1Δ11c standards. GC–MS analysis confirmed these peaks were C18:1Δ9t, C18:1Δ7c, C18:1Δ9c, C18:1Δ11c. [file 12934_2016_431_MOESM5_ESM.pptx]

## Slide 1
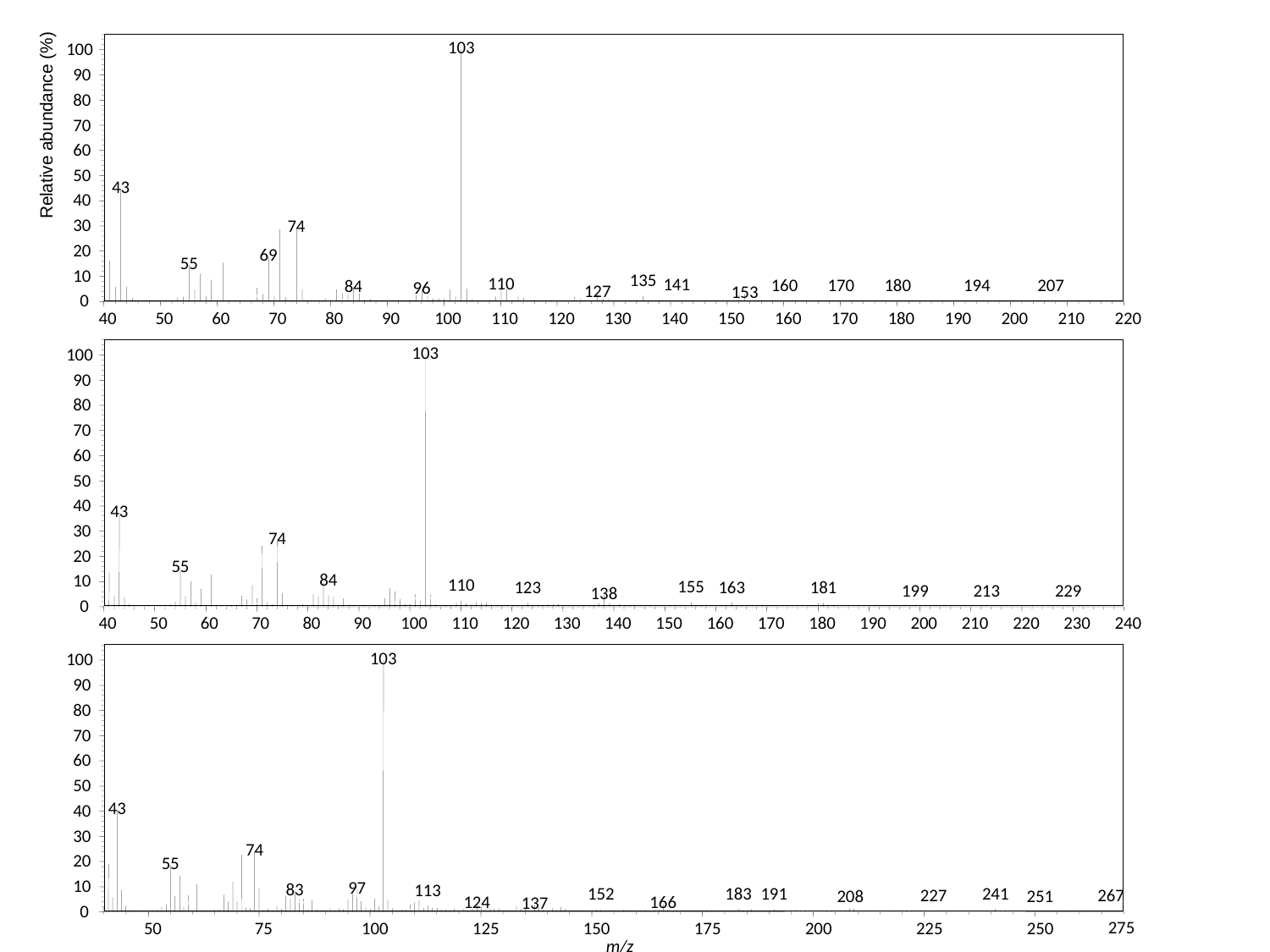

103
100
90
80
70
Relative abundance (%)
60
50
43
40
30
74
20
69
55
10
135
110
141
180
160
170
194
207
84
96
127
153
0
40
50
60
70
80
90
100
110
120
130
140
150
160
170
180
190
200
210
220
103
100
90
80
70
60
50
40
43
30
74
20
55
84
10
110
155
163
181
123
213
229
199
138
0
40
50
60
70
80
90
100
110
120
130
140
150
160
170
180
190
200
210
220
230
240
103
100
90
80
70
60
50
43
40
30
74
20
55
10
97
83
113
241
183
152
191
227
267
208
251
124
166
137
0
275
50
75
100
125
150
175
200
225
250
m/z

Supplement: Supplementary file 6 — 10.1186/s12934-016-0431-9 Mass spectra of 3-hydroxyl fatty acid methyl esters. A. 3OH-C10:0 FAME at 5.025 min (M + 202); B. 3OH-C12:0 FAME at 6.992 min (M + 230); C. 3OH-C14:0 FAME at 9.275 min (M + 258). [file 12934_2016_431_MOESM6_ESM.pptx]

## Slide 1
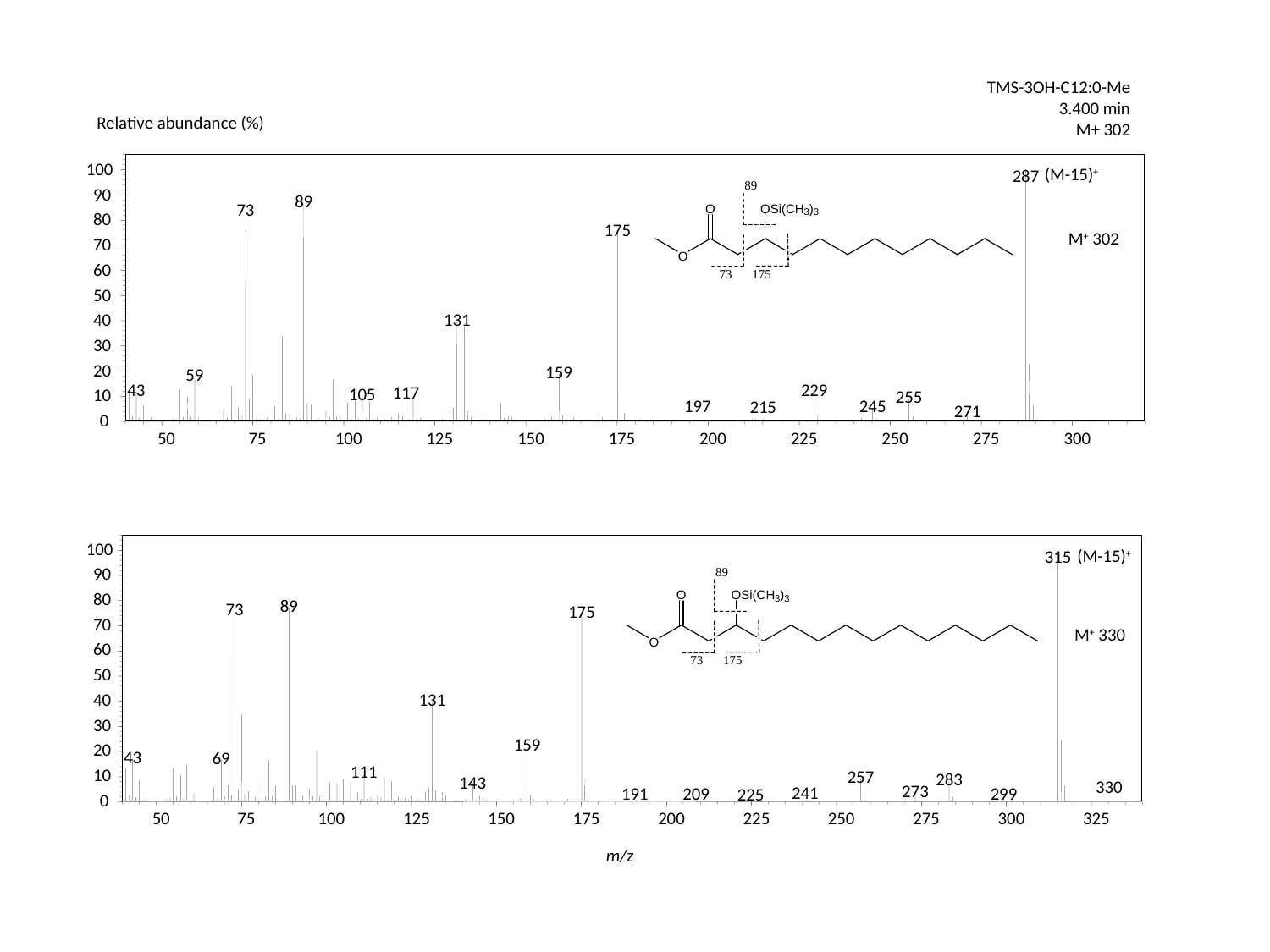

TMS-3OH-C12:0-Me
3.400 min
M+ 302
Relative abundance (%)
(M-15)+
100
287
90
89
73
80
175
M+ 302
70
60
50
131
40
30
20
159
59
43
229
117
105
10
255
197
245
215
271
0
50
75
100
125
150
175
200
225
250
275
300
100
(M-15)+
315
90
80
89
73
175
70
M+ 330
60
50
40
131
30
159
20
43
69
111
10
257
283
143
330
273
241
191
209
299
225
0
50
75
100
125
150
175
200
225
250
275
300
325
m/z

Supplement: Supplementary file 7 — 10.1186/s12934-016-0431-9 Mass spectra of TMS derivative hydroxy fatty acid methyl esters and their TMS derivatives. A. 3OH-C12:0 FAME (M + 302); B. 3OH-C14:0 FAME (M + 330). [file 12934_2016_431_MOESM7_ESM.pptx]

## Slide 1
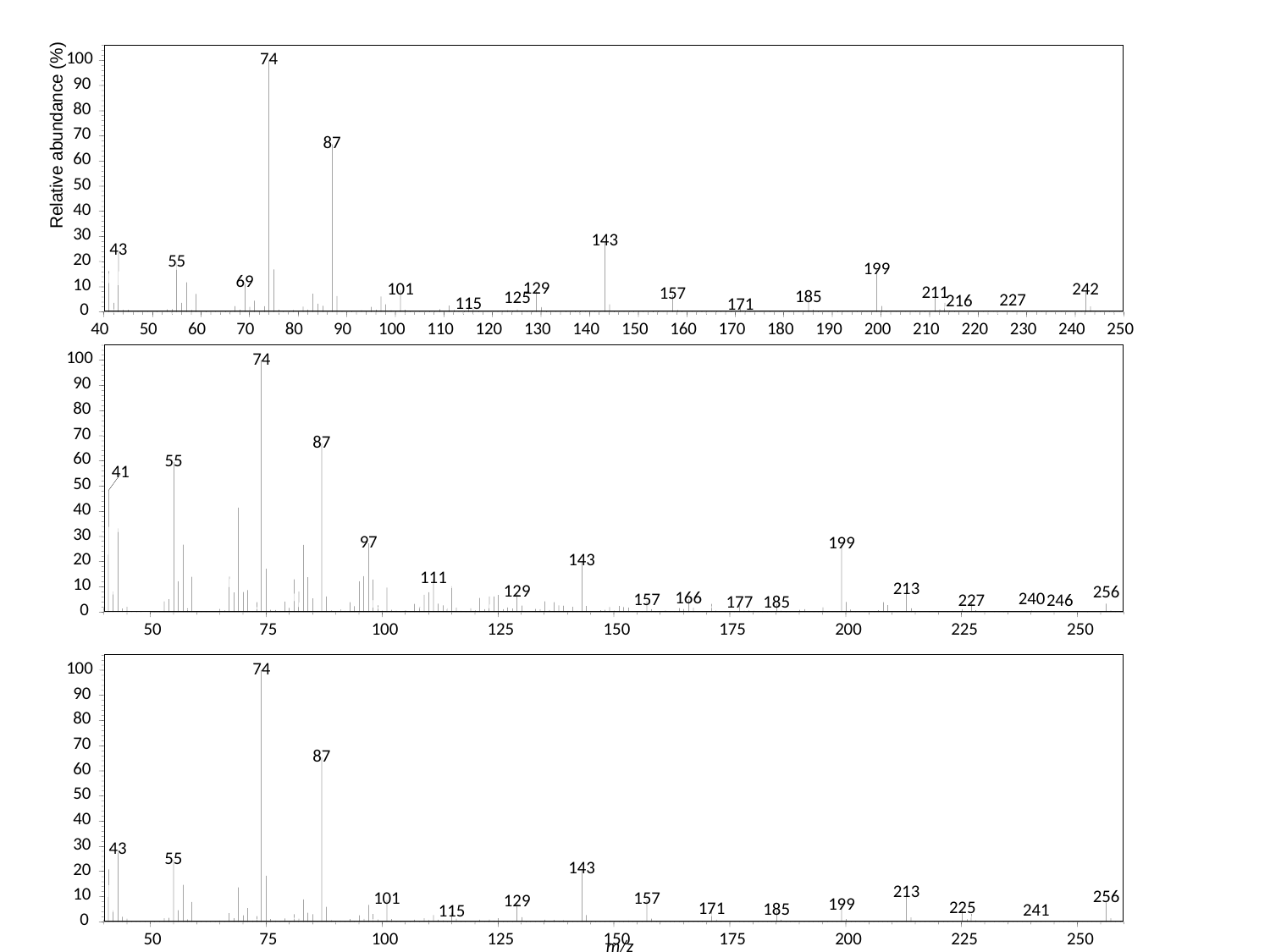

100
74
90
80
70
Relative abundance (%)
87
60
50
40
30
143
43
20
55
199
69
10
129
101
242
211
157
185
125
227
216
115
171
0
40
50
60
70
80
90
100
110
120
130
140
150
160
170
180
190
200
210
220
230
240
250
100
74
90
80
70
87
60
55
41
50
40
30
97
199
20
143
111
10
213
129
256
166
240
157
246
227
177
185
0
50
75
100
125
150
175
200
225
250
100
74
90
80
70
87
60
50
40
30
43
55
143
20
213
10
256
101
157
129
199
225
171
185
241
115
0
50
75
100
125
150
175
200
225
250
m/z

Supplement: Supplementary file 8 — 10.1186/s12934-016-0431-9 Mass spectra of branch chain fatty acid methyl esters. A. C14:0 FAME at 4.075 min (M + 242); B. 12-methyl-C14:0 FAME at 4.600 min (M + 256); C. C15:0 FAME at 4.833 min (M + 256). The different ratio of mass ion between m/z 199 and 213 in B and C suggested B could be the anteiso isomer of C15:0 FAME, i.e., 13-methyl-C14:0. [file 12934_2016_431_MOESM8_ESM.pptx]

## Slide 1
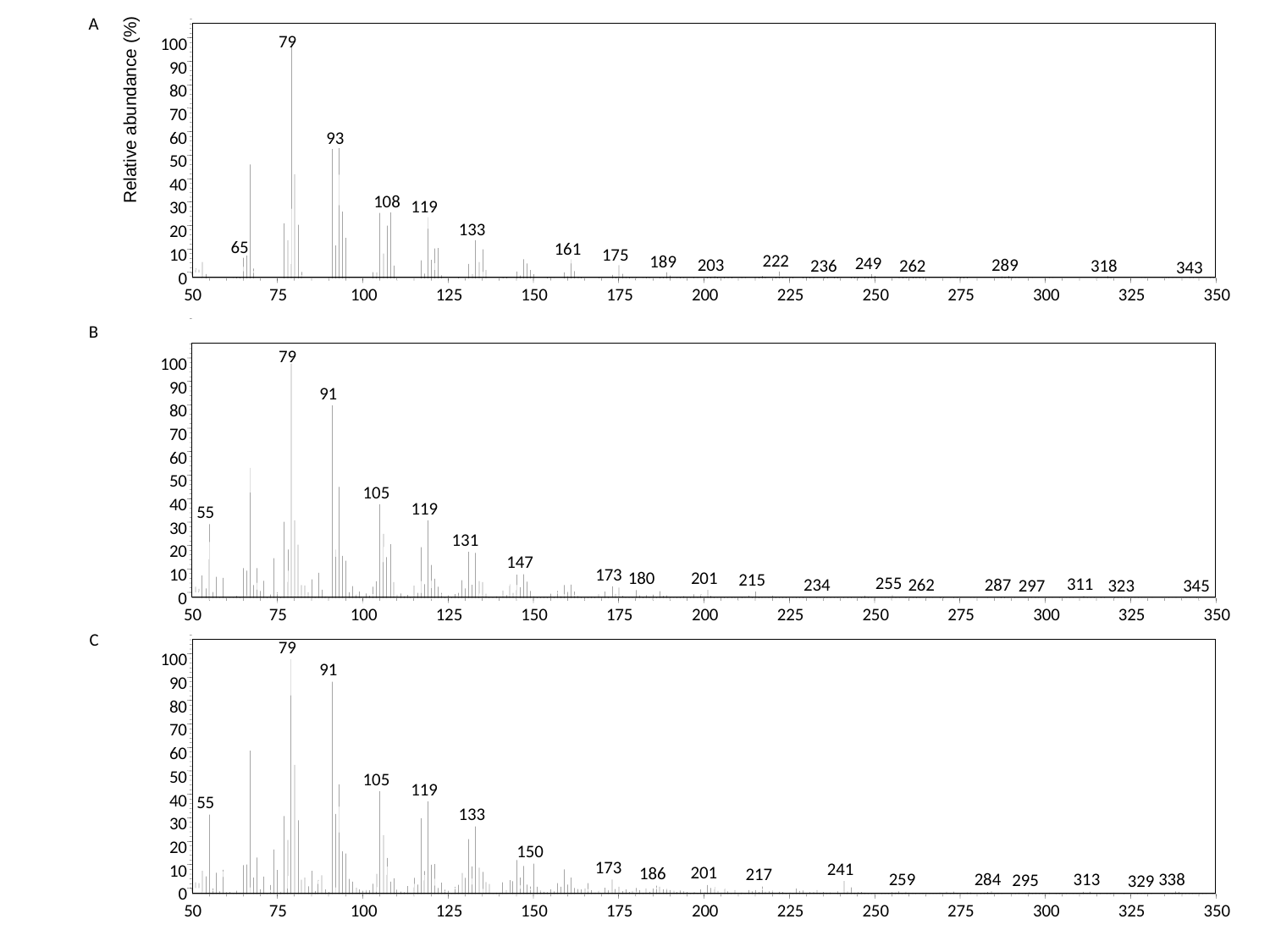

A
Relative abundance (%)
79
100
90
80
70
93
60
50
40
108
119
30
133
20
65
161
175
10
222
189
249
203
289
318
236
262
343
0
50
75
100
125
150
175
200
225
250
275
300
325
350
B
79
100
90
91
80
70
60
50
105
40
119
55
30
131
20
147
173
10
201
180
215
255
311
287
234
262
297
323
345
0
50
75
100
125
150
175
200
225
250
275
300
325
350
C
79
100
91
90
80
70
60
50
105
119
40
55
133
30
20
150
173
241
10
201
186
217
284
338
259
313
295
329
0
50
75
100
125
150
175
200
225
250
275
300
325
350

Supplement: Supplementary file 9 — 10.1186/s12934-016-0431-9 Mass spectra of polyunsaturated fatty acid methyl esters C20:4, C20:5, and C22:5. [file 12934_2016_431_MOESM9_ESM.pptx]

## Slide 1
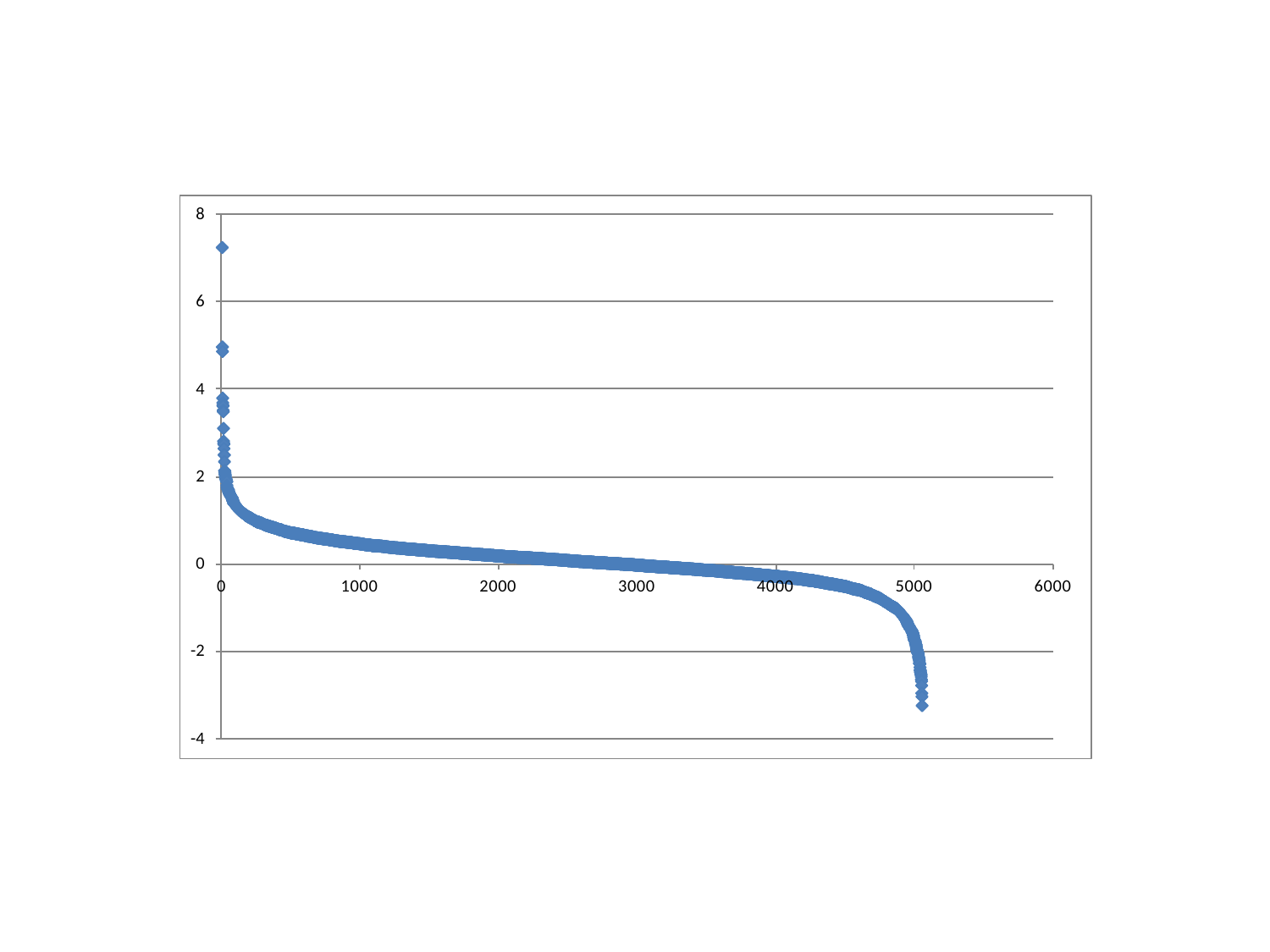

Supplement: Supplementary file 10 — 10.1186/s12934-016-0431-9 Statistical analysis of differential expressed genes after cerulenin treatment. [file 12934_2016_431_MOESM10_ESM.pptx]

## Slide 1
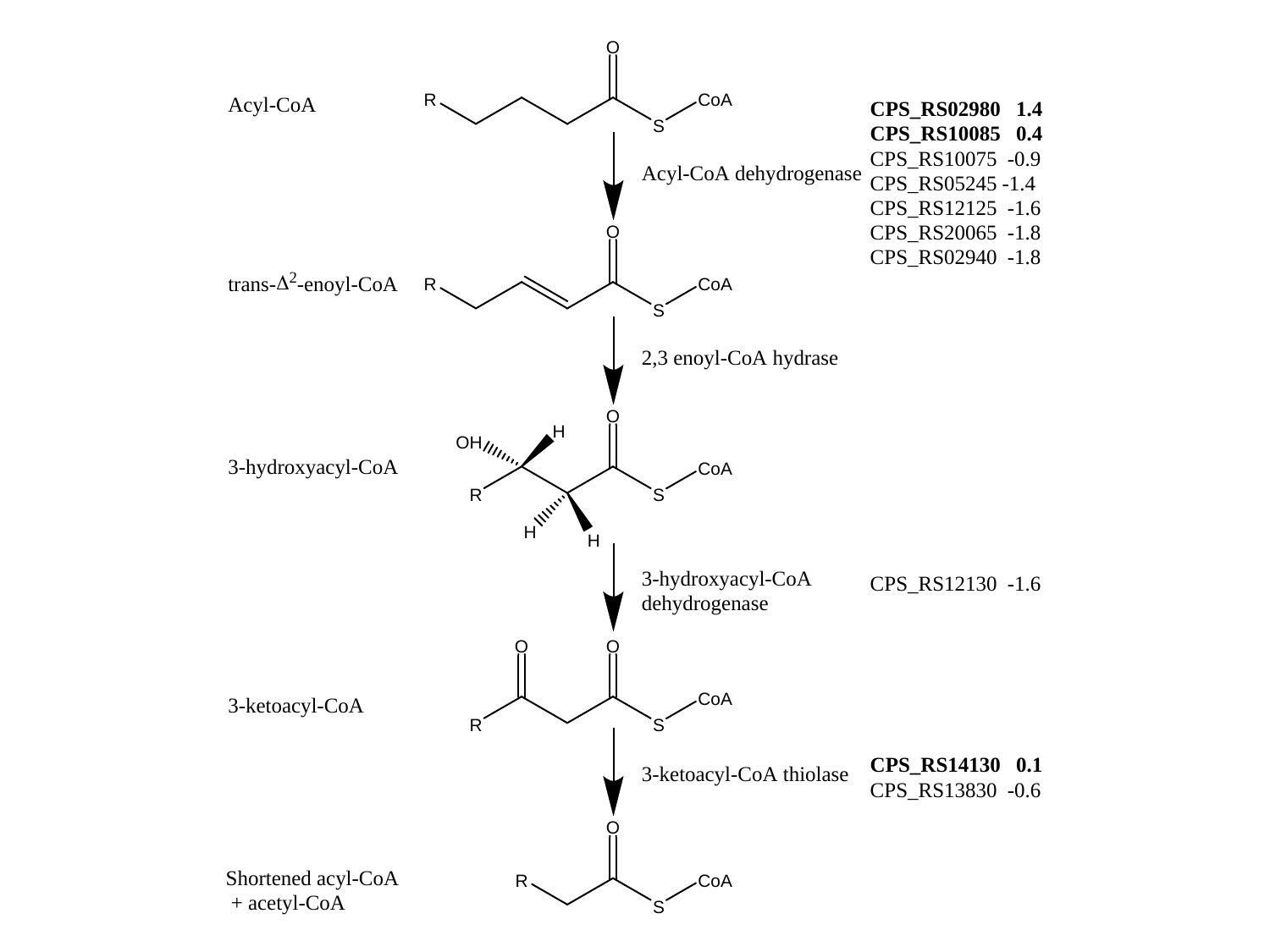

Supplement: Supplementary file 13 — 10.1186/s12934-016-0431-9 Selective down-regulation of oxidation pathway genes. Bold indicated the gene had higher expression level in cerulenin-treated sample (M10_C) than in untreated sample (M10). [file 12934_2016_431_MOESM13_ESM.pptx]
