# Supplementary material for: Effect of cerulenin on fatty acid composition and gene expression pattern of DHA-producing strain Colwellia psychrerythraea strain 34H
Source: Microb Cell Fact. 2016 Feb 6;15:30. doi: 10.1186/s12934-016-0431-9 (PMC4744452; doi:10.1186/s12934-016-0431-9)
Supplement: Supplementary file 11 — 10.1186/s12934-016-0431-9 Analysis of C. psychrerythraea pfa gene expression by RNA-seq and qRT-PCR. [file 12934_2016_431_MOESM11_ESM.docx]

**Additional file 11: Table S3** Analysis of *C. psychrerythraea pfa* gene expression by RNA-seq and qRT-PCR

| Locus ID | Gene ID | Gene description | Fold Change (log_2_) | |
| --- | --- | --- | --- | --- |
|  |  |  | qRT-PCR | RNA-seq |
| CPS_RS13885 | pfaA | polyunsaturated fatty acid synthase | -0.12 | -0.58 |
| CPS_RS13880 | pfaB | polyunsaturated fatty acid synthase | -0.63 | -0.37 |
| CPS_RS13875 | pfaC | polyunsaturated fatty acid synthase | 0.19 | 0.03 |
| CPS_RS13865 | pfaD | polyunsaturated fatty acid synthase | 0.14 | 0.60 |
| CPS_RS13895 | pfaE | 4'-phosphopantetheinyl transferase | -0.20 | -0.51 |
