# Supplementary material for: Effect of cerulenin on fatty acid composition and gene expression pattern of DHA-producing strain Colwellia psychrerythraea strain 34H
Source: Microb Cell Fact. 2016 Feb 6;15:30. doi: 10.1186/s12934-016-0431-9 (PMC4744452; doi:10.1186/s12934-016-0431-9)
Supplement: Supplementary file 12 — 10.1186/s12934-016-0431-9 Differentially expressed genes in C. psychrerythraea treatment without or with 12 μg/mL cerulenin at 10 °C. [file 12934_2016_431_MOESM12_ESM.docx]

**Additional file 12: Table S4** Differentially expressed genes in *C. psychrerythraea*treatment without or with 12 mg/L cerulenin at 10°C

| Gene | Transcript Abundance  (RPKM) | | | | | Fold Change (log_2_) | | | | Gene Description |
| --- | --- | --- | --- | --- | --- | --- | --- | --- | --- | --- |
|  | Without cerulenin | | 12 mg/L cerulenin | | | RNA-seq | | qRT-PCR | |  |
| **Top up-regulated genes** | | | | | | | | | | |
| CPS_RS06205 | | 23.99 | | 3813.25 | 7.26 | | 8.83 | | FMN binding,oxidoreductase | |
| CPS_RS06210 | | 29.44 | | 853.52 | 4.87 | | 7.83 | | unknown | |
| CPS_RS07330 | | 105.41 | | 1415.11 | 3.81 | | 3.71 | | transporter activity | |
| CPS_RS06200 | | 41.77 | | 515.103 | 3.70 | | 5.14 | | TetR family transcriptional regulator | |
| CPS_RS07335 | | 161.30 | | 1922.79 | 3.65 | | 5.40 | | unknown | |
| CPS_RS07340 | | 134.99 | | 1608.74 | 3.63 | | 5.50 | | unknown | |
| CPS_RS07445 | | 22.45 | | 250.87 | 3.53 | | 4.52 | | short chain dehydrogenase/reductase family oxidoreductase | |
| CPS_RS18085 | | 15.02 | | 165.09 | 3.50 | | - | | allantoateamidohydrolase | |
| CPS_RS04135 | | 23.94 | | 192.46 | 3.11 | | 1.67 | | oxidoreductase, zinc-binding | |
| CPS_RS07325 | | 102.74 | | 689.08 | 2.82 | | 4.33 | | TetR family transcriptional regulator | |
| CPS_RS04140 | | 20.32 | | 133.50 | 2.77 | | - | | peptidase activity | |
| **Top down-regulated genes** | | | | | | | | | | |
| CPS_RS05395 | | 691.70 | | 80.47 | -3.10 | | - | | phosphate ABC transporter periplasmic phosphate-binding protein | |
| CPS_RS06270 | | 169.74 | | 21.17 | -3.00 | | -1.87 | | toluene tolerance family protein | |
| pstA | | 131.87 | | 19.48 | -2.76 | | -1.65 | | inorganic phosphate transmembrane transporter activity | |
| acpD1 | | 36.42 | | 238.66 | 2.71 | | - | | FMN reductase activity | |
| CPS_RS17855 | | 468.56 | | 72.18 | -2.69 | | - | | TonB-dependent receptor | |
| CPS_RS19080 | | 58.42 | | 9.45 | -2.62 | | - | | unknown | |
| pstC | | 115.49 | | 19.14 | -2.59 | | - | | transporter activity | |
| CPS_RS17780 | | 279.45 | | 46.39 | -2.59 | | - | | protein transporter activity | |
| CPS_RS17765 | | 333.69 | | 56.51 | -2.56 | | - | | iron ion transmembrane transporter activity | |
| exbB3 | | 370.04 | | 63.95 | -2.53 | | - | | protein transporter activity | |

Fold change (log_2_ values) in transcript levels under specified conditions as determined by RNA-seq. Values above 2.5 or below -2.5 are listed. Transcript abundance obtained from RNA-seq data is indicated as RPKMs (See Methods).
